# Pharmacological Modulators of TCTP: Opportunities and Challenges for Drug Repurposing

**DOI:** 10.3390/ijms27167161

**Published:** 2026-08-11

**Authors:** Weronika Andrzejczyk, Klaudia Porębska, Thananjeyan Balasubramaniyam, Agnieszka Synowiec, Malgorzata Kloc, Paweł Stączek, Jacek Z. Kubiak

**Affiliations:** 1 Military Institute of Medicine—National Research Institute, Szaserów 128, 04-141 Warsaw, Poland; kporebska@wim.mil.pl (K.P.); tbalasubramaniyam@wim.mil.pl (T.B.); asynowiec@wim.mil.pl (A.S.); 2 BioMedChem Doctoral School, University of Lodz, Lodz Institutes of the Polish Academy of Sciences, Jana Matejki 21/23, 90-237 Lodz, Poland; 3 Transplant Immunology, The Houston Methodist Research Institute, Houston, TX 77030, USA; mkloc@houstonmethodist.org; 4 Department of Surgery, The Houston Methodist Hospital, Houston, TX 77030, USA; 5 Department of Molecular Microbiology, University of Lodz, Banacha 12/16, 90-237 Lodz, Poland; pawel.staczek@biol.uni.lodz.pl; 6 Dynamics and Mechanics of Epithelia Group, Institute of Genetics and Development of Rennes (IGDR), Faculty of Medicine, UMR 6290 CNRS, Univeristy of Rennes, 35043 Rennes, France

**Keywords:** TCTP, pTCTP, dTCTP, HRF, fortilin, cancer, sertraline, PLK1

## Abstract

Thanks to the rapid development of advanced analytical methods, researchers can now identify new, so far unknown applications for existing drugs. This expanding knowledge about the mechanisms of drug action, including interactions with various molecules and molecular pathways, not only enables more effective use of available therapies but also reduces both the time and cost of developing new therapeutic options. One of the proteins reported to interact with numerous drugs and chemicals is Translationally Controlled Tumor Protein (TCTP). Numerous studies have demonstrated that TCTP plays a crucial role in diverse biological processes, including cell growth, cell cycle regulation, cellular proliferation, allergic reactions, stress response, inhibition of apoptosis, calcium ion binding, interacting with microtubules and actin microfilaments, the progression of various cancers, and tumor reversion. Therefore, this review article aims to compile a list of drugs and substances shown to interact with TCTP and that, given TCTP’s numerous cellular functions, could be repurposed for other therapies.

## 1. Introduction

TCTP, also known as fortilin, histamine-releasing factor (HRF), and p21/p23, is a protein composed of 172 amino acids [1]. TCTP is encoded by the *Tpt1* gene and is an evolutionarily conserved protein. The structure of human TCTP (hTCTP) consists of three α-helices and eleven β-strands. The eleven β-strands form three β-sheets: β-sheet A-C. β-sheet A connects the N- and C-terminal regions of hTCTP and forms a β-barrel structure with β-sheet B. The short α1 helix inserts into the β-barrel structure. The two longer helices, α2 and α3, run parallel to each other, forming an α-hairpin structure with a short loop that packs onto the β-barrel. Thus, hTCTP has a well-folded core structure containing β-barrel and α-hairpin domains. The hTCTP structure contains two main hydrophobic cores located on opposite sides of the β-sheet B plane. Backbone dynamics analysis reveals that the three domains of hTCTP exhibit distinct dynamic properties, consistent with their structural organization [2].

**Figure 1:**
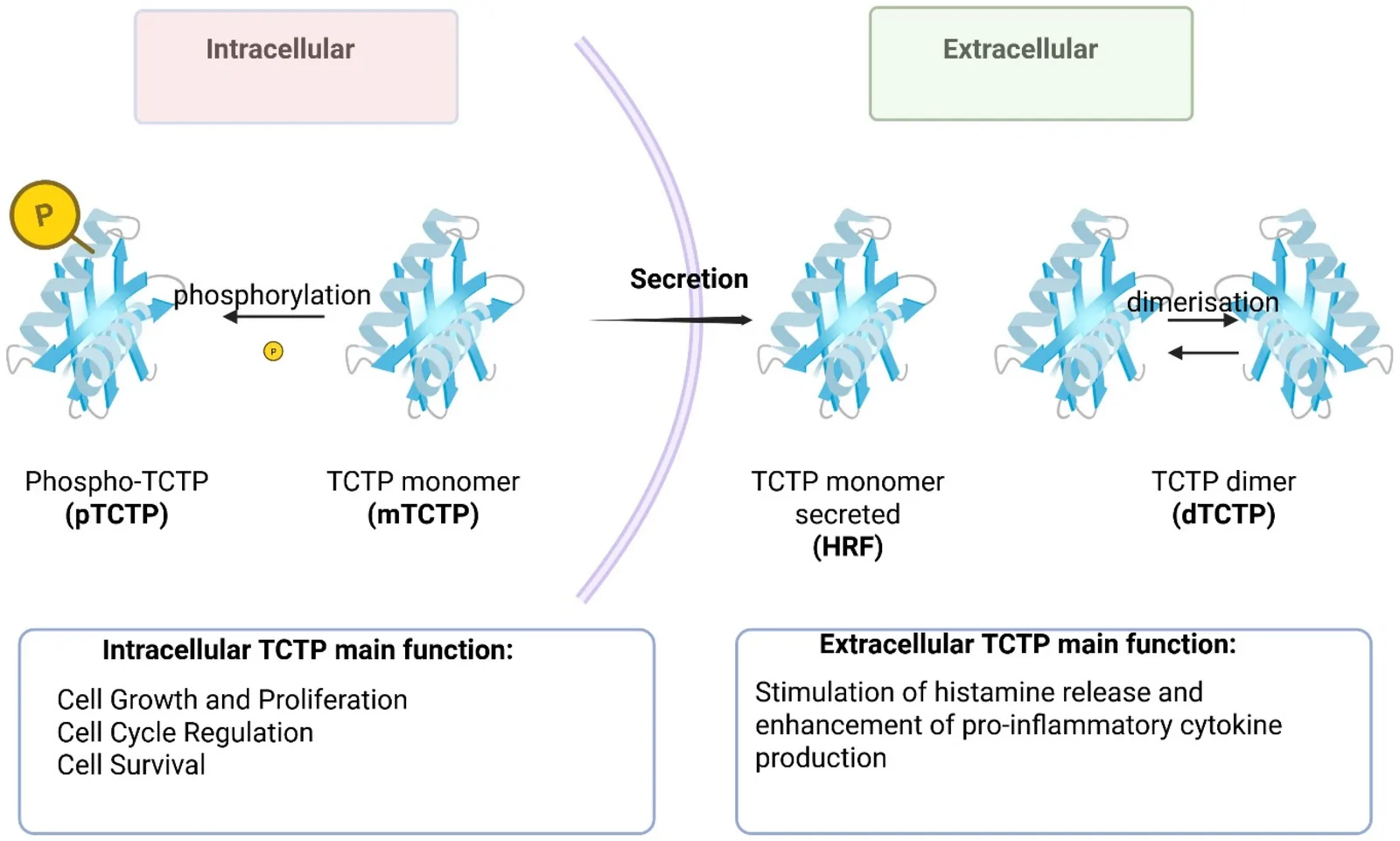
Schematic representation of the intracellular and extracellular forms of translationally controlled tumor protein (TCTP) and their principal functions. Created in BioRender. Brodaczewska, K. (2026) https://BioRender.com/my4m6re.

TCTP is expressed in all eukaryotic organisms. However, its expression levels vary across cell types, tissues, and developmental stages [3]. For example, TCTP is expressed at low levels in postmitotic tissues such as the brain, but at high levels in tissues undergoing active cell division and in tumor cells [4]. Although TCTP is primarily a cytoplasmic protein, nuclear localization has also been reported, and it can be secreted extracellularly [3]. TCTP plays diverse roles in both intracellular and extracellular contexts [5]. A graphical overview is presented in Figure 1.

## 2. Main Functions Performed by TCTP Intracellularly

### 2.1. Cell Cycle Regulation

**Figure 2:**
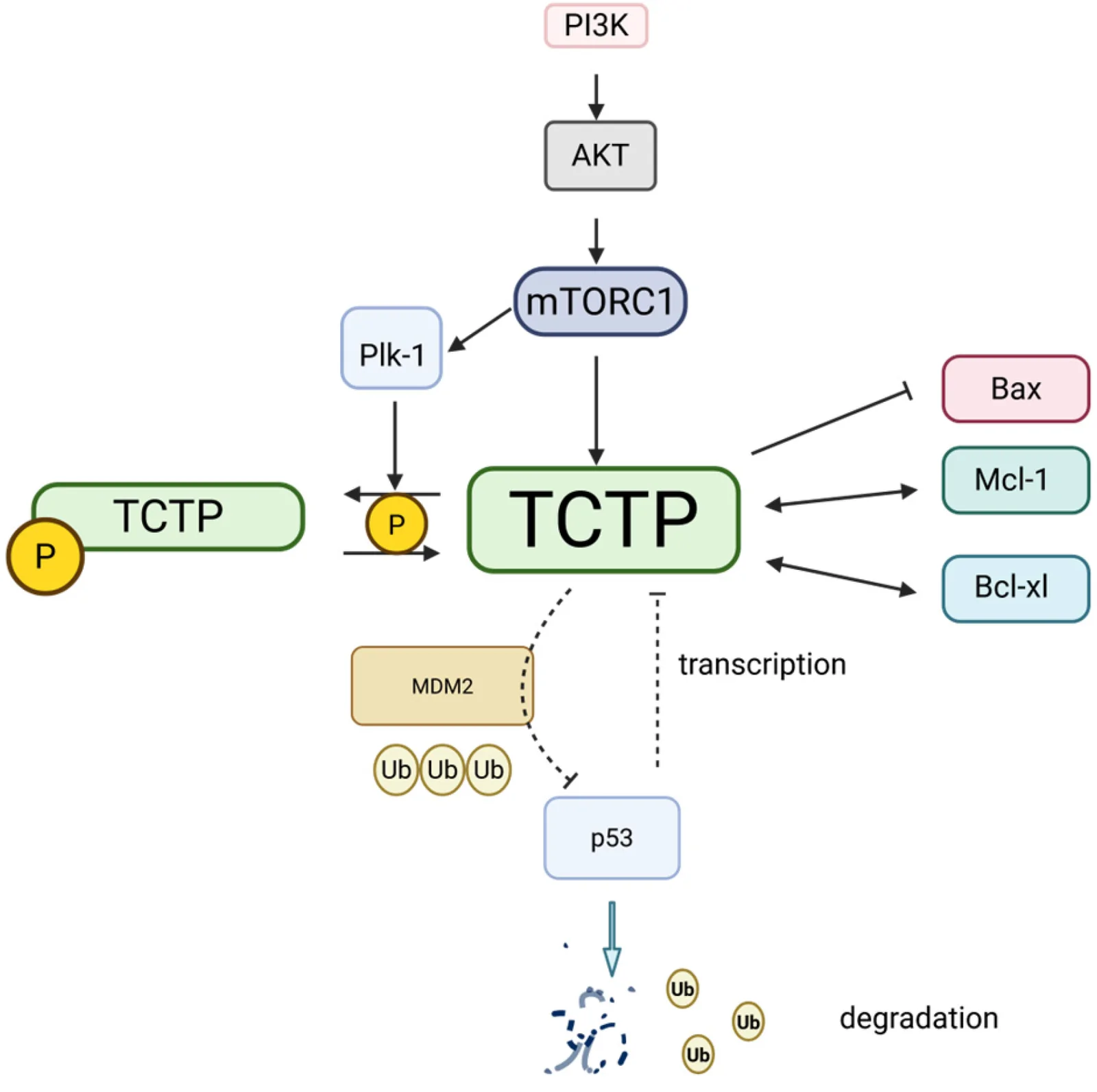
Major signaling pathways involved in TCTP-mediated regulation of cell death. Created in BioRender. Brodaczewska, K. (2026) https://BioRender.com/8lusa4e.

TCTP functions as a tubulin-binding protein. A portion of TCTP remains bound to microtubules throughout most of the cell cycle, including association with the division spindle, but dissociates from the spindle upon metaphase-anaphase transition [3,6]. It also has an affinity for actin microfilaments [7]. Additionally, TCTP promotes the ubiquitin-dependent degradation of p53 (as shown in Figure 2), a tumor suppressor protein essential for coordinating cell cycle checkpoint activation, DNA repair mechanisms, and the initiation of apoptosis and cellular senescence [8,9].

### 2.2. Cell Survival

TCTP promotes cell survival by modulating members of the Bcl-2 protein family: the anti-apoptotic proteins Bcl-xL and Mcl-1, and the pro-apoptotic protein Bax. The N-terminal region of TCTP contains a BH3-like domain that interacts with the BH3 groove of Bcl-xL, and TCTP forms heterocomplexes with Bcl-xL [10]. TCTP increases Mcl-1 stability by reducing its ubiquitinylation, thereby inhibiting its proteasome-mediated degradation [11]. Moreover, a specific TCTP domain competes with Bax by binding to the mitochondrial surface receptor thereby inhibiting Bax-mediated proapoptotic pathways [12]. Finally, TCTP antagonizes apoptosis by inserting into the mitochondrial membrane and inhibiting Bax dimerization [13].

Another mechanism that enhances cancer cell survival, involving the TCTP protein, is the EGFR-AKT-MCL-1/CXCL10 pathway. TCTP binds to the Na^+^/K^+^-ATP pump in a phosphorylation-dependent manner, thereby activating the epidermal growth factor receptor (EGFR), which in turn activates proliferation and survival pathways. This stimulates AKT and inhibits apoptosis. AKT increases the expression of the anti-apoptotic protein MCL-1. At the same time, there is a decrease in CXCL10 (a chemokine that attracts T lymphocytes) [14].

### 2.3. Cell Growth and Proliferation

TCTP is highly conserved across all eukaryotes. It has been shown to control cell growth and proliferation by regulating Rheb GTPase activity in *Drosophila melanogaster*. Rheb (Ras homolog enriched in brain) is a member of the Ras superfamily of GTPases. Researchers have shown that TCTP acts as a guanine nucleotide exchange factor (GEF) for Rheb, a GTPase that directly activates mTOR complex 1 (mTORC1), one of two mTOR protein complexes that regulate growth signaling and protein synthesis. It was demonstrated in *in vivo* studies, where reduced levels of TCTP in *Drosophila* (dTCTP) resulted in decreased cell size, cell number, and organ size, phenocopying the effects of *dRheb* mutations. Furthermore, the activity of dTCTP as a guanine nucleotide exchange factor (GEF) for dRheb was evaluated in vitro using a GDP release assay. While dRheb alone exhibited only weak intrinsic GDP dissociation, the addition of dTCTP markedly accelerated GTP/GDP exchange on dRheb. These findings suggested that dTCTP may function as GEF or a related regulatory factor to activate dRheb, thereby promoting cell growth and proliferation through positive regulation of dRheb activity [15]. However, shortly after these data were published, a study appeared which did not confirm the above conclusions. NMR spectroscopy revealed no direct interaction between TCTP and Rheb. Similarly, studies performed in mammalian cell lines showed that reducing TCTP levels did not affect the activity of ribosomal protein S6 kinase (S6K), a direct downstream effector of Rheb. These findings suggest that TCTP is unlikely to function as a guanine nucleotide exchange factor (GEF) for Rheb [16].

Given the differences among the published studies, we reviewed the available literature investigating the relationship between TCTP and Rheb. To date, there is still insufficient and inconclusive evidence to definitively establish TCTP as a guanine nucleotide exchange factor (GEF) for Rheb. The most recent study addressing this issue, published in 2022 by Frappaolo et al. [17], investigated the role of *Drosophila* Golgi phosphoprotein 3 (dGOLPH3) in the Tctp–Rheb–mTORC1 signaling axis. Although the authors confirmed an interaction between TCTP and Rheb, their findings did not demonstrate that TCTP functions as a Rheb GEF [17].

The question of how TCTP influences mTOR via Rheb remains a point of contradiction, but there is now a growing body of research suggesting that mTORC1 affects TCTP levels.

Studies suggest that the PI3K/Akt/mTORC1 pathway plays a role in the translational induction of TCTP synthesis; furthermore, mTORC1 inhibition decreases intracellular TCTP levels [18].

### 2.4. Autophagy

Studies indicate that TCTP plays a role in regulating autophagy, a key degradation mechanism that maintains cellular homeostasis. TCTP inhibits autophagy by activating the mTORC1 pathway, the main inhibitor of autophagy, and by deactivating the AMP-activated protein kinase (AMPK) pathway, which promotes the initiation of autophagy under energy stress conditions. Conversely, silencing of TCTP enhances autophagy by stimulating both autophagosome formation and maturation [19].

### 2.5. Stress Responses

The pro-survival properties of TCTP were also described in the context of protecting cells against endoplasmic reticulum (ER) stress-induced apoptosis. TCTP interacts with the cytoplasmic domain of IRE1α (the stress sensor), inhibiting its kinase and endoribonuclease (RNase) activities [20].

### 2.6. Metabolic Regulation

In addition to its role in the stress response, TCTP has recently been recognized as a glucose-regulated protein that protects pancreatic β-cells under hyperglycemic conditions. Furthermore, it is essential for the developmental expansion of β-cell mass, as its genetic knockout results in hyperglycemia and glucose intolerance [21].

### 2.7. Ion Transport

TCTP inhibits Na^+^/K^+^-ATPase, leading to Na^+^ and Ca^2+^ accumulation and enhanced intracellular mobilization, which triggers neurotransmitter release, including catecholamines from sympathetic neurons [21]. Furthermore, *in vivo* studies in transgenic mice overexpressing TCTP (TCTP-TG) showed systemic arterial hypertension. It has been suggested that this is due to TCTP overexpression, which inhibits the cytosolic domain of Na, K-ATPase activity, ultimately leading to increased cytosolic Ca^2+^ levels, enhanced vascular contractility, and increased tone [22].

### 2.8. Epithelial–Mesenchymal Transition (EMT)

TCTP induces EMT, a process critical for cancer metastasis and tissue remodeling [23]. Sun et al. (2019) [24] found that TCTP expression level in mammary epithelial cells correlated with cancer progression. TCTP knockdown inhibited EMT development. TCTP may regulate EMT through interactions with p53 and members of the miR-200 family (miR-200a, miR-141, and miR-429), and is recognized as a main suppressor of EMT. They showed that the three miRNAs were down-regulated in TCTP-overexpressing cells and upregulated in TCTP-depleted cells. They supposed that TCTP has no direct interaction with EMT but regulates EMT through p53, which is the target gene of the miRNAs mentioned above [24].

### 2.9. Intercellular Signaling via Small Extracellular Vesicles (sEVs)

sEVs are nanoparticles with a lipid bilayer; they are secreted by, for example, tumor epithelial cells and other cell types, and are involved in regulating the immune response and in intercellular signaling by delivering active molecules such as proteins and nucleic acids [25]. Furthermore, they participate in the formation of the pre-metastatic niche in tumors by transporting oncogenic material. Amson et al. (2024) [26] demonstrated that TCTP is a key regulator of sEV signaling associated with genotoxic stress and the intercellular transport of oncogenic information. They demonstrated that loss of TCTP results in a significant reduction in sEV secretion, and changes in sEV content, including decreased protein and RNA clevels [26].

## 3. Main Functions of the Phosphorylated Form of TCTP

TCTP also occurs in a phosphorylated form. Phosphorylation of TCTP at two serine residues, Ser46 and Ser64, is regulated by Polo-like kinase 1 (PLK1) [27]. PLK1 is an important regulator of mitotic progression and is expressed in proliferating normal tissues but is absent or highly reduced in non-proliferating cells [28]. TCTP phosphorylation is markedly enhanced during the G2/M phase of the cell cycle, when PLK1 is active [29] and plays a functionally important role during cell division [27].

## 4. Main Functions Performed by TCTP Extracellularly

TCTP is exported from cells into the extracellular space, where it is known as histamine-releasing factor (HRF). Extracellular TCTP releases histamine and exhibits cytokine-like activity [5]. HRF activates mast cells and basophils *via* an IgE-dependent mechanism, thus functioning as a cytokine [30]. Studies confirm that HRF is produced by various cells, including vascular endothelial cells, mononuclear cells, alveolar macrophages, platelets, and B and T cells, during late-phase allergic reactions [5]. Building on these findings, growing clinical evidence suggests that TCTP plays a significant role in the pathogenesis of allergies and inflammatory diseases, highlighting the potential of TCTP inhibitors as an anti-allergic therapeutic strategy. Furthermore, a recent study demonstrated that extracellular TCTP released by dying tumor cells promotes tumor growth by recruiting polymorphonuclear myeloid-derived suppressor cells into the tumor microenvironment [31].

While intracellular TCTP is a monomeric 23 kDa form (mTCTP), extracellular TCTP can undergo dimerization (dTCTP) through N-terminal truncation and formation of disulfide bonds between Cys172 residues of two TCTP monomers. Extracellular dTCTP triggers histamine release and increases the secretion of pro-inflammatory cytokines by basophils, mast cells, and epithelial cells [32]. Moreover, only the dimeric form of TCTP, which forms during inflammatory conditions, exhibits cytokine-like activity [33].

## 5. Regulation of TCTP Protein Levels as a Promising Therapeutic Target

Strategies to reduce TCTP expression may be beneficial when combined with anticancer therapy. Evidence suggests that chemical inactivation of TCTP can sensitize cancer cells to DNA-damaging agents. For example, sertraline, a TCTP-interacting drug, enhances UVC-induced apoptosis in MCF-7 cells and increases sensitivity to the DNA-damaging agent etoposide and the DNA repair inhibitor olaparib. These findings highlight TCTP’s significant potential as a therapeutic target to improve chemotherapeutic efficacy in cancer patients with high TCTP expression [8]. Furthermore, TCTP regulates the radiosensitivity of cancer cells. Its overexpression inhibits radiation-induced cell death, while TCTP silencing increases radiosensitivity [34].

Conversely, TCTP, due to its anti-apoptotic and cytoprotective functions, can have a protective role in anti-cancer therapies. For example, Cai et al. (2019) demonstrated a protective role for TCTP in doxorubicin-induced cardiac dysfunction using a cardiomyocyte-specific TCTP-overexpressing mouse model (TCTP-TG mice) [35].

Numerous studies have examined drugs and substances that modulate TCTP protein levels both inside and outside cells, as well as its dimeric form (dTCTP) and phosphorylated form (pTCTP). Given the diverse effects of these compounds, we classify them in this review article into four categories: (1) agents that reduce TCTP levels, (2) agents that increase TCTP levels, (3) agents that alter phosphorylated TCTP levels, and (4) agents that modulate dimeric TCTP levels.

## 6. Methodology

This review was conducted through a comprehensive search of the scientific literature in the PubMed. Original research articles and review papers published in English between 1998 and 2026 were included. Additional publications were identified through manual screening of the reference lists of selected articles (Table 1).

Studies included in this review were those describing direct interactions between pharmacological compounds and TCTP, modulation of TCTP expression or activity, and effects on signaling pathways known to be involved in the regulation of TCTP. Studies not related to the pharmacological modulation of TCTP were excluded.

This review is narrative in nature and does not follow a formal systematic review protocol or include a meta-analysis. However, inclusion and exclusion criteria were applied, and the selected studies were critically evaluated with respect to the type of evidence provided (in silico, in vitro, animal models, and clinical studies) and their relevance to TCTP biology and potential therapeutic applications.

## 7. Agents Lowering TCTP Levels

### 7.1. Antidepressants/Antipsychotics/Antihistamines

Researchers have reported cytotoxic activities of certain antidepressant drugs interacting with TCTP in different tumor models, where autophagy dysregulation plays a key role. For example, sertraline has been shown to either induce or inhibit autophagy depending on the neoplastic context. Moreover, antidepressants can cross the blood–brain barrier (BBB) and exhibit good tolerability, making them attractive candidates for anticancer therapy [36].

#### 7.1.1. Sertraline

It is a commonly prescribed selective serotonin reuptake inhibitor (SSRI) used to treat several psychiatric disorders, including major depressive disorder [37]. Interestingly, sertraline exhibits anticancer activity distinct from its antidepressant properties, inducing apoptosis in cancer cells. Considerable evidence indicates that sertraline modulates TCTP levels in cells, suggesting a causal effect of TCTP diminution.

The group led by Telerman and Amson pioneered the study of sertraline’s effect on TCTP, with numerous subsequent studies confirming this drug’s pro-apoptotic effects in cancer cells [38]. The proposed molecular mechanisms underlying this activity include: activation of caspase-3/7, inhibition of Bcl-2, endoplasmic reticulum (ER) stress, mitochondrial outer membrane permeabilization, disruption of protein synthesis and survival pathways (including mTOR inhibition), and reactive oxygen species (ROS) generation [39].

Studies suggest that direct binding of sertraline to TCTP inhibits the association between TCTP and MDM2. It leads to the upregulation of MDM2 autoubiquitination and, consequently, causes the accumulation of p53 [9]. In addition, a low cellular level of TCTP may reactivate Bax dimerization, an essential process for its pro-apoptotic activity.

A research group demonstrates that the interaction of TCTP with sertraline sensitizes cells (breast cancer MCF-7 cells) to DNA-damaging drugs, such as etoposide and olaparib, and increases UVC-triggered apoptosis. As a mechanism for this action, it has been suggested that this results from impairment of homologous recombination (HR) repair, since a physiological level of TCTP is required for efficient HR processes [8].

One research group evaluated the role of TCTP in melanoma following sertraline treatment. They tested human (MeWo and A2058) and murine (B16-F1 and B16-F10) melanoma cell lines in vitro and employed an in vivo C57BL/6 mouse model. In cell lines, sertraline decreased TCTP levels and inhibited cell viability, colony formation, and cell migration. Molecular analysis revealed that sertraline treatment increased both p53 and caspase-3 levels while decreasing Ki67 expression. In vivo administration of 10 mg/kg sertraline on the fifth day after melanoma cell injection markedly inhibited tumor growth. All effects of sertraline were dose- and time-dependent [40].

In a study on the effect of sertraline on prostate cancer stem cells (PCSCs): PC3, DU145, and LNCaP, the cells were treated with various concentrations of sertraline (1–100 µM) and incubated for 48 h. The researchers observed a range of dose-dependent effects of sertraline. The IC50 value after 48 h was 10 µM. Doses above 10 µM produced similar effects in all the prostate cancer cell lines studied. 48 h treatment with 50 µM sertraline significantly reduced TCTP protein levels. Additionally, the authors demonstrated that sertraline induced cell-cycle arrest in G0 phase and promoted apoptosis and autophagy in a dose-dependent manner. It also increased the expression of autophagy markers such as LC3-II, Beclin1, and ATG5, and increased the level of activated cleaved Poly [ADP-ribose] polymerase 1 (PARP1), indicating DNA damage and apoptosis. They observed the activation of caspase-3, triggering the apoptotic cascade, disruption of iron metabolism, leading to cell death *via* the generation of iron-dependent ROS, reduction in the expression of Aldehyde Dehydrogenase 1 (ALDH1), which is a marker of stem cells, and two epithelial–mesenchymal transition (EMT) markers: transcription factor 8 (TCF8) and lymphoid enhancer-binding factor-1 (LEF1). The researchers also emphasize that oxidative stress may be one of the primary mechanisms by which sertraline acts on PCSC cells. Sertraline induced reductions in glutathione levels and increases in hydrogen peroxide levels and lipid peroxidation in PCSC cells. The drug, administered at a higher dose of 50 µM, also inhibited the migratory capacity of PCSC cells in wound-healing assays and reduced F-actin polymerization; F-actin forms the actin cytoskeleton and plays a key role in cell migration [41].

Given the recent publication of a comprehensive review on the anti-cancer mechanisms of sertraline [39], these mechanisms will not be discussed in detail here. That review article presented the use of sertraline in multiple cell lines and in vivo analyses, with detailed consideration of molecular mechanisms.

#### 7.1.2. Thioridazine

Thioridazine, a phenothiazine derivative antipsychotic, was historically prescribed for schizophrenia, psychotic disorders, and anxiety. Recent evidence suggests that thioridazine inhibits cancer cell growth, although its exact molecular mechanism remains unclear [42].

Amson et al. (2012) [9] showed that thioridazine’s cellular effects may be related to its strong interaction with TCTP and to its inhibition of TCTP function. They confirmed that TCTP increases p53 expression by counteracting MDM2-induced p53 ubiquitination [9].

The same research group [38] observed a correlation between the cytotoxic effects of several drugs (thioridazine, sertraline, perphenazine, chlorpromazine, paroxetine, and flupenthixol) and TCTP inhibition. Notably, upregulation of TCTP mRNA by sertraline and thioridazine observed by them suggests that the transcriptional machinery remains unaffected and may compensate for reduced TCTP protein levels [38].

#### 7.1.3. Sertraline/Thioridazine

Amson et al. (2024) [26] conducted a study using microscale thermophoresis (MST) to investigate the interactions between sertraline, thioridazine, and recombinant human TCTP. For this purpose, they used various concentrations of sertraline and thioridazine, ranging from 0.01 to 200 μM (1:1 dilutions), which were titrated against a constant concentration of TCTP (785 nM). They identified significant binding of thioridazine to TCTP (Kd = 5.39 ± 2.1 μM) and of sertraline to TCTP (Kd = 15.45 ± 1.4 μM), confirming binding of both drugs to TCTP. Furthermore, in a Kaplan–Meier survival analysis of Trp53^−/−^ mice treated orally with sertraline at 30 mg/kg body weight, 5 days a week, starting at 6 weeks of age, compared with untreated Trp53^−/−^ mice, which received no treatment, it was shown that sertraline mimics the effect of TCTP reduction in the Trp53^−/−^ tumorigenesis process in mice. In this study, the survival of mice treated with sertraline was significantly prolonged.

Given the interaction between TCTP and DDX3, as demonstrated by researchers, which affects miRNA content in sEVs, they also demonstrated the effect of sertraline and thioridazine on the formation of the TCTP-DDX3 complex. It showed that thioridazine, and to a lesser extent sertraline, disrupted the formation of this complex. Therefore, these drugs may significantly impact sEV content and the transfer of oncogenic material between cells *via* sEVs [26].

#### 7.1.4. Levomepromazine and Buclizine

Levomepromazine, a phenothiazine derivative, is an antipsychotic (neuroleptic) with sedative, antidepressant, and antiemetic properties. Buclizine, a piperazine derivative, is an antihistamine with antiemetic and anticholinergic effects.

Among 12 antihistaminic compounds tested, including the control drugs promethazine and hydroxyzine, levomepromazine and buclizine exhibited the highest in silico binding affinities to recombinant TCTP, with Kd values of 57.2 μM (*p* < 0.01) and 433 μM (*p* < 0.01), respectively. The researchers also investigated how these compounds affected TCTP expression and cell cycle progression. Seventy-two-hour incubation with levomepromazine or buclizine significantly decreased TCTP protein level, inhibited MCF-7 cell growth, and induced G1 phase cell cycle arrest in a dose-dependent manner. Both drugs significantly decreased the levels of cyclin D1, cyclin D3, CDK2, and CDK4, but did not affect the cyclin-dependent kinase inhibitors p21 and p27 [43].

#### 7.1.5. Buclizine

Another group demonstrated that pharmacological suppression of TCTP protein by buclizine inhibits regeneration in the earthworm *Perionyx excavatus*. They injected *P. excavatus* individuals with buclizine at three dosage levels (140, 160, or 200 µg) into the post-clitellar region (24th segment) once daily throughout the experiment. All buclizine concentrations significantly inhibited regeneration in post-amputated worms on days 1, 4, and 6, reducing regeneration to approximately one-fourth that of controls, with no significant differences among the three dosages. To confirm the interaction between the TCTP protein and buclizine, the researchers performed molecular docking studies, demonstrating that buclizine formed hydrogen bonds and hydrophobic interactions with the *L. rubellus* TCTP protein, with a binding energy of −6.1 kcal/mol [44].

### 7.2. Antimalarials

#### 7.2.1. Artemisinin

Artemisinin is a sesquiterpene lactone extracted from the Chinese herb *Artemisia annua*. Artemisinin and its semisynthetic derivatives possess antimalarial activity [45], and numerous studies have examined the effects of artemisinin and its derivatives on TCTP levels.

Efferth’s group attempted to investigate the exact binding site of artemisinin within TCTP using conventional mass spectrometry (MS). However, they were unable to detect artemisinin-modified peptides by this method. Therefore, the researchers enriched peptides from the N-terminal region of TCTP and identified fragments alkylated by an artemisinin-derived probe. Using an activity-based probe, they characterized an irreversible alkylation mechanism of TCTP by heme-activated artemisinin. A “capture-and-release” approach enabled the identification of TCTP peptide fragments alkylated by artemisinin. However, this approach required introducing an alkyne modification into the artemisinin structure, which could alter the binding mode and mechanism of the artemisinin–TCTP interaction [46,47].

#### 7.2.2. Artesunate

Artesunate is a semi-synthetic derivative of artemisinin. Kobayashi et al. (2014) [48] demonstrated that TCTP levels inversely correlated with sensitivity to artesunate, which significantly suppressed the viability of human malignant peripheral nerve sheath tumor (MPNST) cells, but not normal Schwann cells. Combined treatment with artesunate and rapamycin had a greater effect on sNF96.2 cell viability than either drug alone [48].

#### 7.2.3. Dihydroartemisinin (DHA)

DHA is the main active metabolite of artemisinin derivatives. It is also used as a first-line antimalarial drug in various countries and exhibits low toxicity. Fujita et al. (2008) [49] investigated the relationship between DHA and TCTP. Using a surface plasmon resonance-based method, the researchers demonstrated that DHA binds to human TCTP with a Kd of 38 μM. Upon binding to human TCTP, DHA increases its ubiquitination, shortens the half-life of TCTP in a proteasome-dependent manner, and reduces cellular TCTP levels in various cell types: vascular smooth muscle cells VSMC, lung cancer cell lines A549 and H1299, osteosarcoma cell line U2OS, breast cancer cell lines MDA468 and MCF7, treated with vehicle and 20 μM DHA for 48 h. Instead, transcriptional activation of the TCTP gene was observed. The researchers also found that cells with silenced TCTP expression were less susceptible to DHA-induced apoptosis, whereas cells overexpressing TCTP were more sensitive to DHA than wild-type cells. This suggests that the proapoptotic effects of DHA may be at least partly mediated through TCTP [49].

Liu et al. (2014) also found that DHA inhibits the proliferation of A549 lung cancer cells while upregulating TCTP mRNA expression and downregulating TCTP protein expression [50].

To investigate the role of TCTP in the survival of cardiomyocytes with high TCTP expression levels, Cai et al. (2019) [35] used primary cultures of neonatal rat ventricular myocytes (NRVMs), which were treated with 1 and 2 µM concentrations of DHA for 48 h. DHA treatment induced cardiomyocyte death accompanied by TCTP downregulation. Loss of TCTP protein level led to the death of cardiomyocytes, exhibiting features of apoptosis and autophagy, accompanied by the opening of the mitochondrial permeability transition pore (mPTP), similar to those observed in cell death induced by Bnip3 (Bnip3 is a pro-apoptotic member of the BH3-only subfamily of Bcl-2 family proteins) [35].

Another research group showed that DHA reduced both total and pTCTP protein levels. This DHA-induced reduction in pTCTP induced mitotic aberrations and increased microtubule density in the breast cancer cell lines HCC1954 and HCC1569. DHA concentrations (ranging from 1.25 to 5 µM) that inhibited cell viability within the first 24–48 h also reduced the levels of both total TCTP and pTCTP, as shown by Western blot analysis. Fluorescence analysis showed reduced perichromosomal localization of pTCTP. DHA-treated cells exhibited a significant increase in the number of abnormal mitotic spindle structures, including multipolar and monopolar spindles, as well as improper chromosome positioning. Cell cycle distribution analysis demonstrated that DHA induced a G1/S phase transition block in a small subset of HCC1569 cells. Furthermore, Western blot analysis revealed that DHA treatment increased the levels of cyclin B1 and phospho-histone H3 in HCC1954 cells, suggesting that DHA may delay mitotic progression [51].

Lee et al. (2022) [14] found that TCTP inhibition with DHA sensitizes tumors to T cell-mediated therapy. For example, in anti-PD-L1 therapy, DHA reduces TCTP levels, thereby reducing AKT pathway activation, a process that promotes PD-L1 expression (PD-L1 is a molecule that inhibits the T-cell response). Consequently, DHA synergizes with anti-PD-L1 therapy, leading to more effective T-cell lysis of cancer cells [14].

### 7.3. Immunosuppressants

#### Rapamycin

Rapamycin is a macrolide immunosuppressant that inhibits the mechanistic target of mTOR [52]. Rapamycin inhibits mTORC1 by forming a complex with the FKBP12 protein at a site near the kinase domain of mTOR, but this interaction does not block all mTORC1 functions [18]. Researchers treated the human malignant peripheral nerve sheath tumor (MPNST) SNF96.2 cell line with 100 nM rapamycin for 8 h and found that rapamycin down-regulated TCTP at the protein level, but not at the mRNA level [48].

Another research group assessed the relative rate of TCTP protein synthesis in HeLa cells using a pulsed stable isotope labelling with amino acids (pSILAC) in cell culture. The rate of TCTP synthesis is inhibited by rapamycin by about 50% and by other mTOR inhibitors (PP242 or AZD8055) by about 80%. They also found that TCTP mRNA levels remain unchanged in the presence of mTOR kinase inhibitors [18].

The research group of Jeong et al. (2021) [29] investigated the relationship between rapamycin-induced reductions in mTOR levels and their effects on TCTP. They demonstrated that rapamycin’s effect on TCTP protein levels is not due to the inhibition of translation, but rather that mTORC1 regulates TCTP at the post-translational level, and that mTOR inhibition leads to TCTP degradation via the ubiquitin-proteasome system. Additionally, they demonstrated that rapamycin reduces Mcl-1 expression, and due to the mutual stabilization between TCTP and Mcl-1, thereby reduces the stability of the TCTP protein [29].

### 7.4. Anti-Cancer Drugs

#### 7.4.1. Oxaliplatin

Oxaliplatin is a platinum-based compound that inhibits DNA synthesis primarily by forming intrastrand DNA cross-links and is widely used in clinical chemotherapy [53]. Proteomic screening identified 21 proteins consistently altered by oxaliplatin across three colon cancer cell lines (HT29, SW620, and LoVo). Cells were treated with oxaliplatin at 70, 15, and 10 μM for corresponding durations of 24, 48, and 72 h. As a result of this treatment, oxaliplatin induced S-phase accumulation and G2/M-phase arrest in colon cancer cell lines. Proteomic analysis indicated that TCTP expression increased significantly within the first 24 h, but then gradually decreased from 48 to 72 h in all three cell lines. The authors hypothesize that platinum exposure triggers transient upregulation of TCTP, followed by subsequent downregulation reflecting apoptotic responses. Overall, oxaliplatin modulates TCTP expression, induces cell cycle arrest, promotes apoptosis, and inhibits cell proliferation, thereby exerting anticancer effects [54].

#### 7.4.2. Oxaliplatin or 5-Fluorouracil (5-FU)

Another group found that TCTP protein levels increased in HCT116 colon cancer cells in response to 5-FU and oxaliplatin, whilst TCTP mRNA levels decreased. Treatment of HCT116 cells with increasing concentrations of 5-FU (5, 20, 80 µM) or oxaliplatin (2.5, 12.5, 62.5 µM) for 24 h or 48 h resulted in a significant increase in endogenous TCTP protein levels, with the most pronounced effect observed after 48 h of treatment with higher concentrations of 5-FU. A similar, but less pronounced effect was observed with oxaliplatin application. They explain that the translational regulation of TCTP expression under these conditions outweighs the effect of reduced mRNA levels or protein stabilization. The researchers report that the upregulation of TCTP levels in HCT116 cells after treatment with oxaliplatin or 5-FU is mediated through the mTORC1 signaling pathway. They confirmed this by using two mTOR kinase inhibitors, which resulted in almost complete suppression of the increase in TCTP levels. They also found that reducing TCTP levels *via* TCTP knockdown sensitizes HCT116 cells to 5-FU or oxaliplatin. This indicated that TCTP upregulation is a part of the stress response of colorectal cancer cells to these anticancer drugs [55].

#### 7.4.3. Doxorubicin (DOX)

Doxorubicin is an anthracycline antibiotic that is widely used in cancer therapy. However, approximately 1/10 of patients are reported to suffer cardiac side effects. Cai et al. (2019) [35] indicated a role of TCTP in cardiomyocyte survival and suggested that TCTP can prevent DOX-induced cardiac dysfunction. They also found that doxorubicin treatment markedly suppressed TCTP protein expression in cultured cardiomyocytes and mouse heart tissue. TCTP expression was inhibited in cardiac tissues following both acute and chronic doxorubicin treatment. These findings suggest that reduced TCTP expression may be linked to doxorubicin-induced heart failure (cardiotoxicity). They indicated that, at least in part, Bcl-2/adenovirus E1B 19 kDa interacting protein 3 (Bnip3) (one of the pro-apoptotic members of the BH-3-only subfamily of Bcl-2 family proteins) is a mediator of TCTP-loss-induced cell death caused by doxorubicin. Also, DOX-induced decrease in TCTP expression was attenuated in p53-deficient mice (p53 KO) compared with wild-type (WT) mice, indicating that DOX suppresses TCTP expression at least in part through a p53-dependent mechanism. In this case, the therapeutic aim in preventing doxorubicin-induced cardiotoxicity appears to be to prevent a decrease in TCTP levels in cardiomyocytes [35].

### 7.5. Other Drugs and Compounds

#### 7.5.1. Amentoflavone (AF)

Amentoflavone is chemically known as 8-[5-(5,7-dihydroxy-4-oxo-chromen-2-yl)-2-hydroxyphenyl]-5,7-dihydroxy-2-(4-hydroxyphenyl) chromen-4-one. It is a biflavonoid compound isolated from extracts of *Selaginella tamariscina*. In a study on the effects of amentoflavone on proliferating hypertrophic scar fibroblasts (HSFBs), researchers demonstrated its inhibitory effect on cell viability and observed a series of cellular changes consistent with apoptosis. Cells were stimulated with AF at 50 μM and 100 μM for 48 h, and protein extracts were analyzed by Western blot using specific antibodies. The data indicated significant increases in the levels of several proapoptotic factors, such as cleaved caspases 3, 8, and 9 and Bax, and a significant decrease in the antiapoptotic TCTP. Furthermore, they showed significant inhibition of endothelial cell viability, migration, and tube formation, all of which are associated with angiogenesis [56].

#### 7.5.2. 18β-Glycyrrhetinic Acid (18β-GA)

18β-GA is one of the metabolites of glycyrrhizic acid (GL), a main active compound in Licorice, a commonly used traditional Chinese medicine. A range of biological activities has been demonstrated, primarily due to its properties similar to those of adrenal cortex hormones, and its anti-inflammatory, immunomodulatory, anti-tumor, antioxidant, and hepatoprotective effects. A recent study suggested that 18β-GA induces apoptosis in hepatocellular carcinoma (HCC) cells by triggering cell cycle arrest, activating caspase-dependent pathways, and activating the PPARγ pathway [57]. The research conducted by Zhang et al. (2025) has shown that the effect of 18β-GA in inhibiting proliferation, arresting the cell cycle, and reducing metastasis in gastric cancer (GC) cells may be linked to the TCTP protein. Firstly, molecular docking analyses predicted binding of 18β-GA to TCTP, yielding a binding energy of −8.56 kcal/mol. This suggested that 18β-GA may effectively bind to the TCTP protein. Experiments using GC cell lines have shown that following treatment with 18β-GA, there was a significant reduction in the expression of TCTP, p-AKT, and Ki67, whilst P53 expression increased. Furthermore, treatment with 18β-GA significantly increased the levels of Bax, P27, P21, and E-cadherin, whilst simultaneously reducing the levels of Bcl-2, cyclin D1, N-cadherin, and vimentin. As a result, researchers suggest that 18β-GA may promote cell apoptosis by inhibiting the TCTP/AKT/P53 signaling pathway, arrest the cell cycle progression, and inhibit the proliferation and metastasis of gastric cancer [58].

#### 7.5.3. Nutlin-3a

Nutlin-3a is a small molecule that binds to the p53-binding pocket of MDM2, thereby inhibiting the p53-MDM2 interaction and reducing p53 degradation, which activates cell cycle arrest and apoptosis. Researchers have demonstrated that Nutlin-3 also inhibits the TCTP-MDM2 interaction both in vitro and ex vivo setting, thereby highlighting an additional mechanism by which Nutlin-3 suppresses MDM2 function [59].

Nutlin-3a indirectly promotes TCTP degradation by activating p53, exploiting the antagonistic relationship between TCTP and p53. In their research on the earthworm *P. excavatus*, Rajagopalan et al. (2023) [44] demonstrated that worms injected with Nutlin-3a show an inhibition of the regeneration process. Impaired regeneration following administration of Nutlin-3a was also associated with a decrease in the levels of proteins such as: Proliferating Cell Nuclear Antigen (PCNA), which is associated with cell proliferation; Wnt3a, which is associated with stem cell activation; and Yes-associated protein 1 (YAP1), which is associated with the cell growth control (Hippo signaling pathway), whilst increasing p53 expression [44].

#### 7.5.4. Tanshinone IIA (Tan-IIA)

Tan-IIA is a bioactive compound from *Salvia miltiorrhiza* that exhibits anticancer activity by inhibiting TCTP protein expression. Tanshinone IIA downregulates TCTP and reduces the expression of the anti-apoptotic proteins Bcl-xL and Mcl-1, while increasing the expression of the pro-apoptotic proteins Bax, caspase-9, and caspase-3, thereby inducing apoptosis. These changes accelerate cancer cell apoptosis and inhibit gastric cancer cell growth and proliferation [1].

#### 7.5.5. Sann-Joong-Kuey-Jian-Tang (SJKJT)

SJKJT is a traditional Chinese medicine that is prescribed as complementary therapy for solid tumor patients in Taiwan. Chien et al. (2013) demonstrated that SJKJT inhibited BxPC-3 cell proliferation, induced apoptosis, and increased the accumulation of cells in the subG1 phase. SJKJT upregulated Bax, caspase-9, TNF-α, caspase-8, and caspase-3, while downregulating Mcl-1, Bcl-2, Bcl-xL, and TCTP. These effects were dose- and time-dependent, with a reduction in TCTP observed after 72 h of treatment with 0.6 mg/mL SJKJT [60].

Similar studies were conducted by Chen et al. (2013) [61], who investigated the effects of SJKJT on the human hepatic cell carcinoma cell line HepG2. HepG2 cells were treated with SJKJT (1 mg/mL) for different durations (0, 24, 48, and 72 h), and the protein expression levels of Fas, caspase-8, caspase-3, Bcl-xL, Mcl-1, TCTP, and Bax were evaluated. The results revealed that increasing the treatment duration of SJKJT decreased the protein expression levels of Mcl-1 and TCTP after 72 h treatment, but increased the protein expression levels of Bcl-xL, Fas, caspase-8, caspase-3, and Bax. These results demonstrated that SJKJT effectively induces apoptosis in HepG2 cells. The molecular mechanisms may involve downregulation of TCTP and Mcl-1 protein expression and upregulation of Fas, TNF-α, caspase-8, caspase-3, and Bax expression [61].

#### 7.5.6. Curcumin

Curcumin (diferuloylmethane), extracted from *Curcuma longa* rhizomes, exhibits preventive and therapeutic effects against various cancers through multiple mechanisms [62]. Cheng et al. demonstrated that curcumin inhibits proliferation and induces apoptosis in HepJ5 cells by triggering endoplasmic reticulum stress and mitochondrial dysfunction. This mitochondrial dysfunction was associated with reduced protein expression of TCTP, Mcl-1, and Bcl-2. They tested various concentrations of curcumin (0, 3, 6, 15, 30, and 60 μM) over different durations (24 and 48 h), and the effect was both dose- and time-dependent [63].

#### 7.5.7. Plasminogen Activator Inhibitor-1 (PAI-1)

PAI-1 is an anticancer agent that inhibits plasmin-driven proteolysis, thereby limiting angiogenesis and metastasis. PAI-1 belongs to the SERPIN family, primarily known for its role in regulating fibrinolysis. However, it is now known that this inhibitor contributes to numerous (patho)physiological processes, including inflammation, wound healing, cell adhesion, and tumor progression [64]. Jankun et al. (2007) found that PAI-1 treatment downregulated nucleophosmin and TCTP protein expression [65].

**Figure 3:**
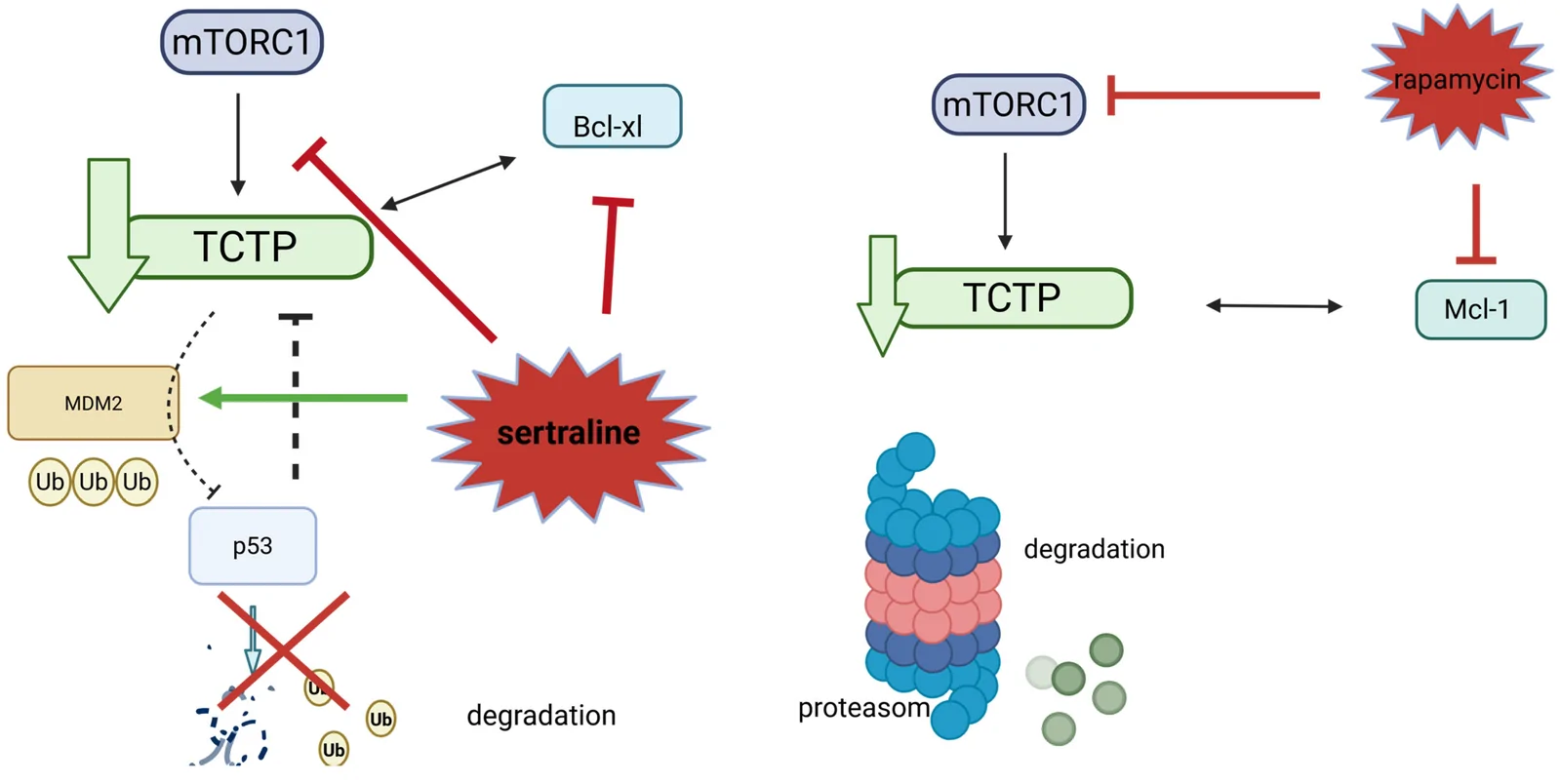
Representative mechanisms of action of drugs that downregulate TCTP: sertraline and rapamycin. Created in BioRender. Brodaczewska, K. (2026) https://BioRender.com/hyrucnv; https://BioRender.com/1wbwf82.

Summary of the Mechanisms Underlying TCTP Downregulation and Interactions Between TCTP and TCTP-Lowering Drugs (Table 2, Figure 3).

Available evidence indicates that only a subset of TCTP-lowering compounds directly interacts with the TCTP protein. These include sertraline, thioridazine, artemisinin and its derivative dihydroartemisinin (DHA), buclizine, and, according to in silico studies, 18β-glycyrrhetinic acid (18β-GA). In contrast, the remaining compounds discussed either reduce TCTP levels indirectly or have not yet been investigated for their interaction with TCTP.

TCTP downregulation may occur through several mechanisms, including suppression of gene transcription, inhibition of mRNA translation, or proteasome-mediated degradation of the protein. Despite extensive research, identifying the primary mechanism underlying TCTP downregulation remains challenging.

In many cases, the decrease in TCTP expression appears to result from the combined action of multiple mechanisms. For example, sertraline may inhibit the mTOR signaling pathway, thereby reducing TCTP translation, and affect mitochondrial proteins such as Bcl-2 as well as modulates the p53 regulatory pathway. Similarly, rapamycin suppresses mTOR signaling, leading to reduced TCTP protein levels without affecting TCTP mRNA expression; however, other studies indicate that TCTP degradation occurs post-translationally via the ubiquitin–proteasome system. An example of a compound that decreases TCTP levels primarily through proteasome-mediated protein degradation is dihydroartemisinin (DHA).

## 8. Medications/Substances Affecting Extracellular dTCTP and Secretion of TCTP (Table 3)

### 8.1. Meclizine

Meclizine is a first-generation antihistamine that acts as a non-selective H1 receptor antagonist, helping relieve common symptoms such as dizziness, nausea, and vomiting [33]. In this study, researchers found that meclizine interacts directly with dTCTP and inhibits dTCTP-induced inflammatory responses in the BEAS-2B bronchial epithelial cell line. Furthermore, they found that meclizine directly interacts with dTCTP and attenuates ovalbumin (OVA). It induced airway inflammation in five-week-old female BALB/c mice by reducing immune cell infiltration, cytokine release, and dTCTP expression. In conclusion, meclizine has potential as an antiallergic agent through its inhibition of dTCTP [33].

**Table 3:** The list of Pharmacological agents affecting extracellular dTCTP.

Compound	Effect on TCTP Binding Direct/Indirect	TCTP Level		Level of Evidence	Experimental Model	Potential Molecular Impact of Treatment	Effect of Action	References
		Protein	mRNA					
Extracellular dTCTP								
Meclizine	Direct	Decrease dTCTP	-	in vitro; in vivo	Human bronchial epithelial cell line BEAS-2B; Five-week-old female BALB/c mice.	Inhibition of IL-8 secretion by suppression of JNK and NF-κB signaling pathways activated by dTCTP.	Reduction in immune cell infiltration, cytokine release, and dTCTP expression.	[33]
Extracellular secretion of TCTP								
Pantoprazole/omeprazole	Indirect	TCTP secretion reduced	-	in vitro; in vivo	Human embryonic kidney 293 cell line; HEK293 and human histiocytic lymphoma cell line U937 cells; BALB/c mice.	Suggests that the translocation exporting TCTP from cells could be regulated *via* the electrochemical gradient maintained by H^+^/K^+^-ATPase.		[66]

“-” no information.

### 8.2. Omeprazole and Pantoprazole

Omeprazole and Pantoprazole are drugs that inhibit the secretion of hydrochloric acid in the stomach by blocking the H^+^/K^+^-ATPase enzyme. Choi et al. (2009) [66] found that omeprazole and pantoprazole inhibit TCTP secretion in a drug concentration-dependent manner in HEK293 and U937 cells. The researchers proposed that the translocation apparatus that exports TCTP from cells may be regulated by the electrochemical gradient maintained by the H^+^/K^+^-ATPase [66].

## 9. Agents Increasing TCTP Levels

### 9.1. Lipopolysaccharides LPS

LPS is a component of the cell wall of Gram-negative bacteria and a potent activator of the inflammatory response. LPS increased the amount of mTCTP and dTCTP and the secretion of dTCTP by mast cells—bone marrow-derived mast cells (BMMCs); however, the TCTP transcript level remained unchanged. This suggests that LPS-induced changes in TCTP protein levels may be regulated at the post-transcriptional, translational, and post-translational levels rather than at the transcriptional level. Moreover, the researchers observed that secreted dTCTP significantly increased the expression of IL-8 and pro-inflammatory cytokines in airway epithelial cells [67].

### 9.2. Macrophage Colony-Stimulating Factor (M-CSF)

M-CSF was reported to promote the synthesis and secretion of HRF (TCTP) in mouse peritoneal macrophages [30,68].

### 9.3. H_2_O_2_

H_2_O_2_ was shown to stimulate secretion of HRF (TCTP) by BEAS-2B human bronchial epithelial cells (HBECs) [30,69].

### 9.4. 4b-Phorbol 12-Myristate 13-Acetate (PMA), Forskolin, and Heavy Metals (Copper, Nickel, and Cobalt)

PMA, forskolin, and heavy metals (copper, nickel, and cobalt). Schmidt et al. (2007) [4] indicated that all of these substances induced TCTP expression in 24 h in two cell lines studied at the mRNA and protein levels. The highest induction rate was observed upon the addition of copper [4].

### 9.5. Dioxin, 2,3,7,8-Tetrachlorodibenzo-P-Dioxin (TCDD)

TCDD is the most toxic compound in the dioxin group, which are ubiquitous environmental pollutants. Dioxins are classified as endocrine disruptors and cause a variety of toxic effects, including immunotoxicity, hepatotoxicity, teratogenicity, and tumor promotion. Researchers examined the TCDD dose-dependency of HRF expression in two cell lines: TT2 embryonic stem (ES) cells and mouse embryonic fibroblasts (MEFs). ES cells and MEFs were treated with various doses of TCDD (0, 1, 10, 100, or 1000 nM) for 2 h. HRF mRNA in ES and MEF cells was induced by TCDD in a dose-dependent manner. Furthermore, HRF was induced by 1 µM TCDD in MEF cells in a time-dependent manner with treatments at 0, 2, 6, or 24 h.

The researchers suggested that an aryl hydrocarbon receptor (AhR)-mediated pathway is involved in TCDD-induced HRF expression and may influence HRF mRNA levels. To examine whether TCDD could induce HRF protein secretion, they stimulated human lymphocyte-derived cell lines BJAB and Jurkat with 1 µM TCDD and observed increased HRF protein secretion in the culture media. To clarify whether TCDD can induce histamine release in vivo, they injected TCDD into C57BL/6 mice. Results showed that only 25% of the mice (n = 12) exhibited a 2–3-fold increase in serum histamine levels compared with control mice receiving DMSO. These data suggest that environmental pollutants, such as TCDD, may increase the incidence of allergic disorders by inducing HRF [70].

### 9.6. Hypoxia

Hypoxia—reduced oxygen concentration to 1% O_2_. As demonstrated by one research group, reducing oxygen levels in in vitro culture of the colorectal cancer cell lines LoVo and HCT116 increased both extracellularly secreted and intracellular TCTP levels. The authors analyzed protein levels by Western blot, comparing samples collected during hypoxic culture at 1, 3, 6, 9, and 12 h to a control group measured at time zero. However, the study did not present TCTP levels at the corresponding time points under normoxic conditions [71].

Summary of the Mechanisms and Interactions of Drugs That Increase TCTP Protein Levels (Table 4).

Although the mechanisms of action of individual compounds that increase TCTP levels are the least well characterized in the available literature, a shared feature of all active agents in this group is their toxicity. This suggests that the observed increase in TCTP following in vitro exposure of cells to these agents may represent a stress-response effect.

## 10. TCTP Phosphorylation Inhibitors

### Onvansertib

Onvansertib is a Plk1-specific ATP-competitive inhibitor that blocks the phosphorylation of Plk1 substrates. TCTP and dual-specificity phosphatase Cdc25 are substrates of Plk1. Inhibition of Plk1 by onvansertib reduces phosphorylation of these two substrates. Onvansertib represents a relevant treatment for head and neck squamous cell carcinoma resistant to cisplatin and radiotherapy [72].

In 2020, the research group led by Zeidan et al. (2020) [73] published the results of a Phase Ib clinical trial of onvansertib in combination therapy (with low-dose cytarabine (LDAC) or decitabine) for patients with relapsed or refractory acute myeloid leukemia. They investigated the effect of onvansertib on changes in the level of phosphorylated TCTP (pTCTP) protein relative to total TCTP protein by incubating cells from the human promyelocytic leukemia cell line HL-60 with 1 mL of plasma from patients treated with onvansertib (12–60 mg/m^2^) in combination with LDAC or decitabine for 1 h. The cells were then washed, lysed, and analyzed by Western blotting. The pTCTP/TCTP ratio was measured in cells incubated with plasma collected on day 1 before drug administration and after administration (tmax), and then normalized against the pre-treatment sample.

They observed that the decrease in pTCTP levels parallels the inhibition of PLK1 by onvansertib. They confirmed that pTCTP was reduced by plasma samples taken during treatment, but not by plasma samples in which onvansertib was not detected [73].

**Figure 4:**
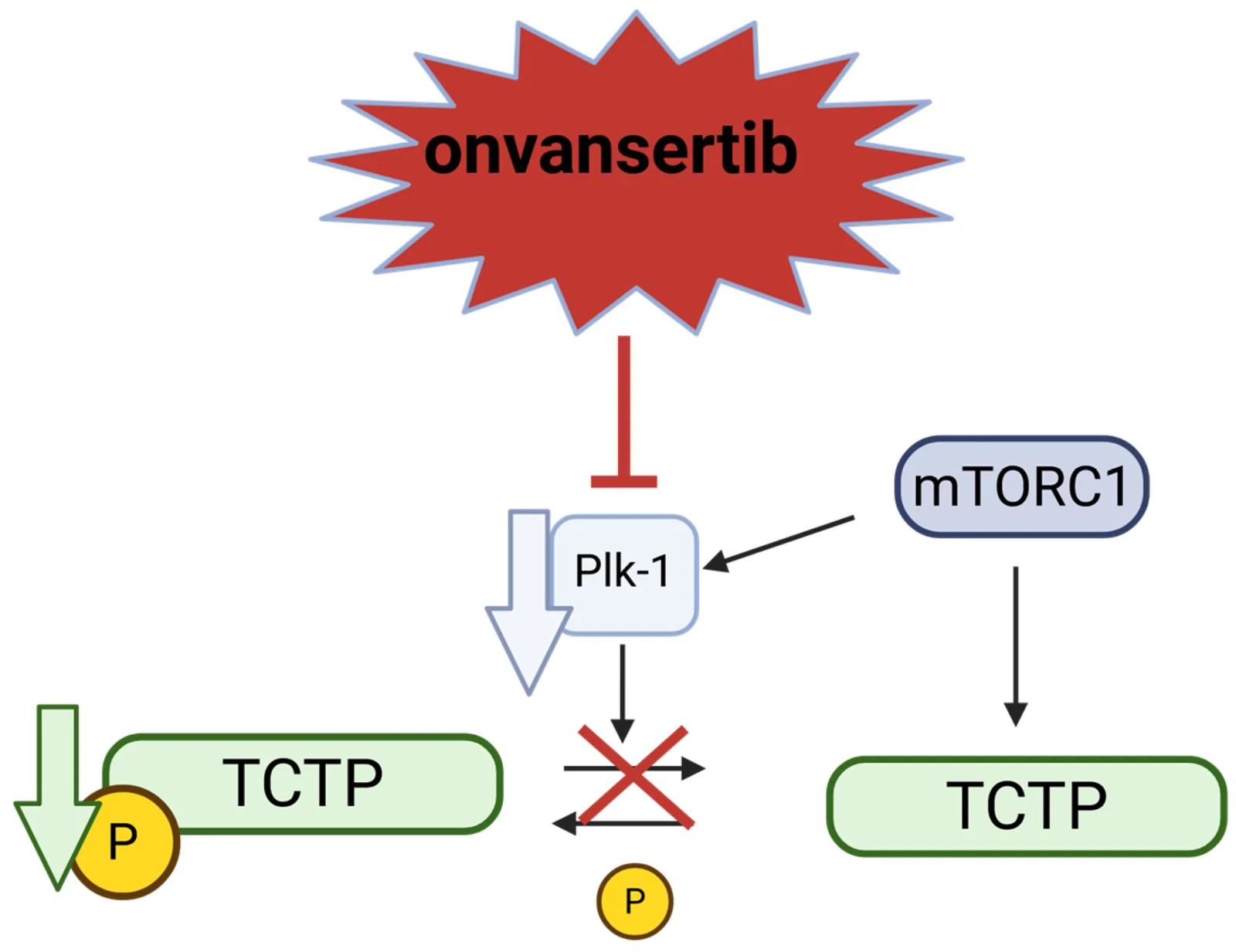
Representative mechanisms of action of drugs that downregulate pTCTP: onvansertib. Created in BioRender. Brodaczewska, K. (2026) https://BioRender.com/hrmhiey.

Summary of Phospho-TCTP-Lowering Drugs (Table 5, Figure 4).

Among the studies reviewed, only one drug, onvansertib, has been shown to reduce phospho-TCTP levels independently of a reduction in total TCTP expression. This effect results from the inhibition of PLK1, the kinase responsible for TCTP phosphorylation. The available evidence supporting this mechanism is based on both in vitro and ex vivo studies.

## 11. Clinical Perspectives

As most of the substances listed in this article are currently used in various therapies, a key question is how to combine the current use of these drugs with their potential new role in other therapies, which may involve modulation of TCTP protein levels.

The best-studied of these drugs is sertraline, a commonly prescribed drug for treating several psychiatric disorders, including major depressive disorder. The recommended maximum daily dose for humans is 200 mg (Summary of Product Characteristics, SmPC https://www.ema.europa.eu/en/glossary-terms/summary-product-characteristics). In the context of research on the use of sertraline in in vivo studies conducted on mice, the administration of sertraline at a dose of 10 mg/kg body weight five days after the injection of cancer cells significantly inhibited tumor growth and reduced TCTP protein levels in the tumors, as confirmed by Western blot analysis [40]. Calculated for an average adult body weight of 70 kg, this would correspond to a dose of 700 mg. These data show that the doses of sertraline that could be effective as an adjuvant in cancer therapy are higher than those recommended for the treatment of depression. However, there are published data on the use of higher doses, ranging from 250 mg to 650 mg per day [74].

It is important to note that between 2004 and 2007, a clinical trial was conducted to assess the effect of sertraline on depression, anxiety, fatigue, reduced quality of life, and survival in patients with advanced cancer. The study involved 189 patients with advanced cancer, most of whom had previously undergone several cancer treatments, particularly chemotherapy, within 30 days before random allocation to groups. However, one of the inclusion criteria was that the patients did not have severe depression diagnosed by physicians. Patients received 50 mg of sertraline once daily (n = 95) or a placebo (n = 94). The trial was stopped before the planned 450 participants had been enrolled, as the possibility of a significant clinical benefit from sertraline use ruled out. No improvement in symptoms, well-being, or survival was observed in patients with advanced cancer who did not suffer from major depression [75]. The cited clinical trial may weaken the arguments in favor of the synergistic use of sertraline in combination with standard anticancer treatment. However, it should be noted that the sertraline dose used (50 mg/day) was relatively low compared to the experimentally tested doses in vitro and in vivo, in mice.

While these clinical studies investigated the off-target effects of sertraline in the context of its potential anticancer therapeutic application, they did not assess TCTP protein levels.

For other drugs, such as levomepromazine and buclizine, the tested concentrations at which a significantly decreased TCTP expression was observed in MCF-7 cells after 72 h treatment (10–25 μM levomepromazine or 60–75 μM buclizine) were within the range of plasma-achievable levels for levomepromazine, but exceeded the maximal buclizine concentration typically reached in human plasma under therapeutic conditions [43].

Another example of a drug with potential as an adjuvant therapy in cancer treatment is dihydroartemisinin. In vitro studies showed a mild but significant inhibition of cell viability within the first 24–48 h at clinically achievable doses [51].

In line with the growing number of in vitro studies demonstrating the potential of DHA as an anticancer agent, clinical trials are being initiated to define the role of DHA as an adjunct therapy in cancer treatment. An example is the Phase II study titled “Icotinib Combined With Dihydroartemisinin (DHA) Therapy in Patients With Advanced Gradually Progressed NSCLC After First-line Icotinib: an Open-label, Single-arm, Multicenter Phase II Study” (NCT03402464). It aims to evaluate the antitumor efficacy of the combination of DHA and icotinib in patients with EGFR-positive non-small cell lung cancer (NSCLC).

However, there are also several limitations in anticancer research on DHA. Notably, neurotoxicity and ototoxicity observed in preclinical studies cannot be overlooked. Therefore, in the future, in addition to developing new dosing regimens, efforts should also focus on reducing DHA-related toxicity and adverse effects [76,77].

Furthermore, in the context of clinical analyses and clinical trials, TCTP/pTCTP is also being investigated for its role as a pharmacodynamic marker, rather than as a therapeutic target on its own. An example of this is the clinical trial: ‘Onvansertib in Combination With Either Low-dose Cytarabine or Decitabine in Adult Patients With Acute Myeloid Leukemia (AML)’, NCT03303339. In this trial, a decrease in pTCTP levels was expected to occur in parallel with the inhibition of PLK1 by onvansertib [73].

TCTP protein levels depend on both tissue type and developmental stage; however, as demonstrated by the studies reviewed, they can also be regulated pharmacologically. Although TCTP overexpression has been shown to exert a protective effect in doxorubicin-treated cardiomyocytes, it may also contribute to chemoresistance. Indeed, in vitro studies have demonstrated that adenovirus-mediated TCTP overexpression protects HeLa cells against cytotoxic agent-induced cell death, including paclitaxel and etoposide [78].

In light of the current evidence that modulating TCTP levels influences therapeutic efficacy, the most promising strategy appears to be the rational design of combination therapies that integrate targeted modulation of TCTP expression with other established therapeutic approaches.

However, a key aspect remains the selection of an appropriate dose, release profile, and site of active-substance delivery, as well as a formulation ensuring optimal pharmacokinetic properties and precise drug targeting. The rapidly advancing field of drug delivery systems currently offers increasingly sophisticated strategies for reducing adverse effects, including cytotoxicity. One example of such an innovative approach is a magnetic nanocomposite hydrogel designed for bidirectionally controlled drug release mediated by an external magnetic field, which could serve as an alternative remotely controlled drug carrier [79].

The use of appropriate vectors or carriers that enable precise drug delivery is equally important given TCTP’s pleiotropic functions in cells. As numerous examples discussed in this review show, TCTP may be an important therapeutic target in cancer, as its downregulation can promote apoptosis and reduce tumor cell proliferation. However, it should be emphasized that TCTP also plays an essential role in the normal functioning of healthy cells, and systemic reduction in its levels could significantly disrupt physiological homeostasis.

Therefore, the path toward the clinical application of the described therapeutic approaches remains limited by challenges related to selecting an optimal dose that is both effective and non-toxic, as well as by the need for precise drug delivery strategies. Importantly, evidence from multiple studies included in this review regarding the effects of clinically used drugs on TCTP levels also provides valuable insight into potential adverse effects and the consequences of drug overdose in patients.

## 12. Significance and Reproducibility of Results

The growing interest in the application in drug repurposing has led many research groups to investigate the effects of previously described drugs on various cellular and molecular processes. However, the results obtained often differ among research centers. Studies concerning sertraline provide an example of such variability. These differences arise from the use of different experimental models (different cell lines derived from various cancer types) or animal models. Nevertheless, even within the same cell line, such as the human breast cancer cell line MCF-7, publications report different IC50 values for sertraline concentrations. For example, one study reported an IC50 of MCF-7 = 25 μM (24 h treatment) [80], whereas another research group reported an IC50 of MCF-7 = 2.45 μM (48 h treatment) [81]. This raises concerns about the reproducibility of research findings across individual research centers. However, the use of different analytical methods for assessing drug toxicity—the sulforhodamine B assay [80] and the MTT assay [81]—as well as different durations of cell exposure to the drug, may justify the differences in the proposed doses. Furthermore, most research groups identify sertraline’s primary activity profile as pro-apoptotic and anti-proliferative.

Discrepancies in the available data also concern the effect of oxaliplatin on TCTP. A PubMed search identified two studies investigating the impact of oxaliplatin on TCTP expression. Both were in vitro studies using established cell lines.

Immunoblot analysis of TCTP expression showed increased protein levels 24 h after initiation of oxaliplatin treatment. The fold change relative to untreated controls (set at 1.0) was 2.73, 3.59, and 2.21 in HT29, SW620, and LoVo cell lines, respectively. At later time points (48 and 72 h), TCTP levels decreased; however, they remained elevated compared with controls. The corresponding fold changes were 1.69, 3.19, and 1.56 at 48 h, and 1.66, 1.45, and 1.42 at 72 h for HT29, SW620, and LoVo cells, respectively [54].

The second research group reported an increase in TCTP protein levels at 24 h, with a more pronounced increase at 48 h; however, they also observed a reduction in TCTP mRNA levels. Although the two studies reported divergent results at the 48 h time point (a decrease relative to 24 h exposure in one study versus a further increase in the other), this discrepancy may be explained by mechanistic differences proposed by one of the groups, suggesting that TCTP upregulation is mediated via the mTOR pathway [55]. At first, stress induced by cytostatics activates mTOR-dependent translation, increasing TCTP protein levels. However, at a later stage, TCTP levels may decrease due to stress-related factors that inhibit mTOR, which, as shown in one study, may be activated at different rates across cell lines [82].

## 13. Conclusions

Numerous studies have demonstrated that TCTP protein levels are highly sensitive to pharmacological modulation. The observed effects depend on both the type of compound used and its mechanism of action, suggesting complex regulation of TCTP expression and stability at the post-translational level.

Although the effects of the described drugs confirm that TCTP may participate in key signaling pathways involved in cell survival, proliferation, and cellular stress responses, and that its levels can be modulated by compounds affecting pathways such as mTOR, p53, Mcl-1, Bcl-xL, or Bnip3, detailed information regarding the signaling pathways involved is still lacking. Therefore, the precise mechanisms underlying the effects of the described substances on TCTP remain unclear.

Selected drugs that reduce TCTP levels show promising potential for future cancer therapies, as TCTP reduction correlates with decreased cell viability. However, the detailed mechanisms of interaction between these drugs/compounds and TCTP remain to be elucidated. Furthermore, the currently available evidence is still derived mainly from basic research, including in silico and in vitro studies. Most of the conducted studies have focused on evaluating the effects of specific compounds on selected cell lines. Among the investigated models, established cell lines derived primarily from different types of cancer have predominated, while only a limited number of studies have been performed using primary cell cultures. This may influence the obtained results, as experimental models may initially exhibit different TCTP protein levels depending on the proliferation rate and biological characteristics of the cell type.

In particular, it should be considered that cancer cell lines generally express higher levels of TCTP protein compared with non-cancerous cells; therefore, these studies may not accurately reflect the effects of the tested compounds in normal cells. Currently, there are no clinical studies investigating the effects of the tested compounds on the modulation of TCTP protein levels as a therapeutic target. This highlights the need for further in vivo studies and, importantly, clinical trials to determine whether these drugs can be translated into new therapeutic applications targeting mechanisms involving TCTP modulation.

Our review also demonstrates how fragmentary the currently available information is about the pharmacological regulation of TCTP, its expression, functions, and posttranslational modifications, showing the necessity of further studies.

## Figures and Tables

**Table 1:** Literature search strategy.

Criteria	Description
Database	PubMed
Language	English
Keywords	TCTP, fortilin, HRF, dTCTP, pTCTP, TCTP decrease, TCTP increase, pharmacological modulation of TCTP
Inclusion criteria	Studies describing the effects of compounds/drugs on the levels of TCTP, pTCTP, dTCTP, or HRF, including investigations involving sertraline-TCTP, artemisinin-TCTP, DHA-TCTP, rapamycin-TCTP, oxaliplatin-TCTP, and onvansertib-TCTP
Exclusion criteria	Studies reporting changes in TCTP, pTCTP, dTCTP, or HRF levels mediated by non-pharmacological approaches, such as CRISPR, siRNA, or shRNA
Types of evidence	In silico, in vitro, in vivo

**Table 2:** Summary of drugs and pharmacological compounds that decrease TCTP expression or activity.

Compound	Effect on TCTP Binding Direct/Indirect	TCTP Level		Level of Evidence	Experimental Model	Potential Molecular Impact of Treatment	Effect of Action	References
		Protein	mRNA					
Antidepressants/antipsychotics/antihistamines:								
Sertraline	Direct	Decrease	Decrease	in vitro in vivo	Human melanoma cell lines (MeWo and A2058); murine melanoma cells (B16-F10); C57BL/6 mice	Increase in P53 and caspase-3 levels and a decrease in TCTP and Ki67 levels.	Inhibition of cell viability, colony formation, and cell migration.	[40]
		Decrease	-	in vitro	Prostate cancer stem cells (PCSC): PC3, DU145, and LNCaP; Human Umbilical Vein Endothelial Cells (HUVEC)	Decrease in TCTP, phospho TCTP protein level; increase expression of LC3-II, Beclin1, and ATG5, cleaved PARP1, activation of caspase-3, reduced expression of ALDH1, TCF8, LEF1; disruption of iron metabolism, generation of iron-dependent ROS, activation of free radical generation, hydrogen peroxide formation, and lipid peroxidation (LPO), along with depletion of glutathione (GSH) levels.	Induction of cell cycle arrest in the G0 phase, induction of apoptosis, and autophagy; reduction in angiogenic potential.	[41]
		Decrease	-	in vitro	Human breast cancer cell line MCF-7	The 24 h of 2.5 μM sertraline treatment significantly decreased TCTP protein level in the treated cells.	Increased apoptosis upon DNA damage and sensitization to etoposide and olaparib.	[8]
Thioridazine/sertraline	Direct	Decrease	-	in vitro	Human colon cancer cell line HCT116	Increased P53 expression by counteracting the MDM2-induced ubiquitination of P53; inhibition of TCTP function.	Provoked apoptosis in tumor cells.	[9]
		Decrease	-	in vitro in vivo	Microscale thermophoresis; Trp53^−/−^ mice	Significant binding of both drugs to TCTP—thioridazine, with a Kd value of 5.39 ± 2.1 μM, and sertraline with a Kd value of 15.45 ± 1.4 μM.	Prolonged survival of Trp53^−/−^ mice treated with sertraline.	[26]
Thioridazine/sertraline/perphenazine/chlorpromazine		Decrease	Upregulation	in vitro	Human monocytic leukemia cell line U937; Human breast cancer cell line MDA-MB-231	Upregulation of TCTP mRNA, but a decrease in TCTP protein level.	Cytopathic effect.	[38]
Levomepromazine/buclizine	Direct	Decrease	-	in silico; in vitro	In silico molecular docking studies; Human breast cancer cell line MCF-7	Levomepromazine bound with a Kd of 57.2 μM and buclizine with a Kd of 433 μM to recombinant TCTP; a significant decrease in TCTP protein level and in cyclin D1, cyclin D3, CDK2, and CDK4.	Inhibition of MCF-7 cell growth, G1 phase cell cycle arrest.	[43]
Buclizine	Direct	Decrease	-	in vivo; in silico	Earthworm, *Perionyx excavatus*; molecular docking	Hydrogen bonding and hydrophobic interactions of *L. rubellus* TCTP protein with buclizine, binding energy of −6.1 kcal/mol; high expression of p53 and lower expression of TCTP in buclizine and thymidine-treated samples.	Inhibition of regeneration in post-amputated worms (approximately one-fourth that of controls).	[44]
Antimalarials:								
Artemisinin	Direct	-	-	in silico	Mass Spectrometry Analysis and Molecular Docking.	Alkylation of multiple amino acids: Phe12 -Tyr22 in TCTP.		[46]
Artesunate	Direct	Decrease	-	in vitro	Malignant peripheral nerve sheath tumors (MPNST) cell line sNF96.2	Inverse correlation with artesunate sensitivity and TCTP level—the drug binds to and degrades TCTP.	Suppression of the viability of MPNST cells, but not normal Schwann cells.	[48]
Dihydroartemisinin (DHA)	Direct	Decrease	Upregulation	in vitro	Human osteosarcoma cell line U2OS, vascular smooth muscle cells VSMC; lung cancer cell lines A549 and H1299, and breast cancer cell lines MDA468 and MCF7.	Direct binding to human fortilin (Kd = 38 μM); increased TCTP ubiquitination, shortening of its half-life, reduction in cellular TCTP protein levels, transcriptional activation of the TCTP gene.	Apoptotic effects induced by DHA treatment.	[49]
		Decrease	Upregulation	in vitro	Lung cancer cell line A549	Upregulation of TCTP mRNA expression, downregulation of TCTP protein expression.	Inhibited cell proliferation of A549 lung cancer cells.	[50]
		Decrease	-	in vitro	TCTP-overexpressing rat cardiomyocyte cells- NRVMs	Enhancement of Bnip3 protein expression in the mouse heart.	Induction of cardiomyocyte death.	[35]
		Decrease	-	in vitro	Mouse colon carcinoma cell line CT26 (P3); human breast adenocarcinoma cell line MDA-MB-231; human melanoma cell line 526Mel; human colorectal carcinoma cell line HCT116; pancreas primary tumor cell PDC110526	TCTP inhibition with DHA sensitizes tumors to T cell-mediated therapy by reducing activation of the AKT pathway, which promotes expression of PD-L1 (a molecule that inhibits the T-cell response).	The synergy between DHA and anti-PD-L1 therapy leads to more effective lysis of cancer cells by T cells.	[14]
		Decrease	-	in vitro	Trastuzumab-resistant breast cancer cells HCC1954 and HCC1569	Decrease TCTP and pTCTP protein level; increase in Cyclin B1 and phospho-Histone-H3.	Inhibition of cell viability. Mitotic aberration and increased microtubule density.	[51]
Rapamycin Indirect		Decrease	No changes	in vitro	Malignant peripheral nerve sheath tumors (MPNST) human cell line sNF96.2	Suppression of mTOR signaling. Down-regulation of TCTP protein but not TCTP mRNA.	The combined use of artesunate and rapamycin increased the cytotoxic effect against MPNST cells.	[48]
		Decrease	No changes	in vitro	pSILAC method, which measures *de novo* rates of protein accumulation in the human cervical cancer cell line HeLa	mTORC1 increases TCTP protein synthesis by regulating the translation of TCTP mRNA. Inhibition of serum-induced increase in TCTP levels in HeLa cells by approximately 50% by rapamycin and even more by mTOR-KIs.		[18]
		Decrease	No changes	in vitro; in vivo	Human lung cancer cell lines: A549, H1299, and H157; immortalized normal human bronchial epithelium cells BEAS-2B; A549 cells xenografted in a mouse model.	mTOR inhibition leads to ubiquitin-proteasome-mediated degradation of TCTP; decreased Mcl-1 expression, and reduced TCTP protein stability due to reciprocal stabilization between TCTP and Mcl-1.	Enhanced efficacy of DNA-damaging drugs, such as cisplatin and doxorubicin, through the induction of apoptotic cell death mediated by TCTP-induced proteolysis.	[29]
Anticancer drugs								
Oxaliplatin	Indirect	Increase at 24 h; decrease at 48 h and 72 h		in vitro	Human colon cancer cell lines: HT29, SW620, and LoVo	Increase in TCTP levels 24 h after oxaliplatin administration, followed by a decrease between 48 and 72 h.	Induction of apoptosis, cell cycle arrest, and inhibition of cell proliferation.	[54,55]
Oxaliplatin or 5-fluorouracil (5-FU)	Indirect	Increase	Decrease	in vitro	Human colon cancer cell line HCT116	Increased TCTP protein levels but decreased TCTP mRNA levels. Increased TCTP protein expression in the presence of 5-FU or oxaliplatin is dependent on mTORC1.	TCTP-siRNA was more sensitive to 5-FU or oxaliplatin. Also, TCTP is indeed able to partially protect HCT116 cells against the cytotoxic effects of 5-FU or oxaliplatin.	[55]
Doxorubicin (DOX)	Indirect	Decrease	Decrease	in vitro; in vivo	Cultured cardiomyocytes; primary cultures of neonatal rat ventricular myocytes (NRVMs); neonatal rat cardiac fibroblasts (NRCFs); mouse heart tissue TCTP transgenic mice (TCTP TG) with cardiac-specific overexpression of TCTP; p53-deficient mice (p53 KO)	Significant suppression of TCTP protein and mRNA expression in cultured cardiomyocytes and mouse heart tissue. DOX suppressed TCTP expression at least in part through a p53-dependent mechanism.	Cardiotoxicity.	[35]
Other substances								
Amentoflavone	Indirect	Decrease	-	in vitro	Proliferating hypertrophic scar fibroblasts (HSFBs)	Increase in the levels of cleaved caspases 3, 8, and 9, and a significant decrease in TCTP.	Significant inhibition of endothelial cell viability, migration, and tube formation.	[56]
18β-Glycyrrhetinic acid (18β-GA)	Direct (In silico *analysis*)	Decrease	-	in vitro; in silico	Human gastric cancer (GC) cell lines: AGS (CL-0022) and MKN-45 (CL-0292); Molecular docking technology.	The binding energy of 18β-GA-TCTP is −8.56 kcal/mol. Reduction in expression of TCTP, p-AKT, Ki67, Bcl-2, cyclin D1, N-cadherin, and vimentin proteins, with increased expression of P53, Bax, P27, P21, and E-cadherin proteins.	Induction of cell cycle arrest; inhibition of proliferation and metastasis of GC cells in vitro.	[58]
Nutlin-3a	Indirect	Decrease	-	in vivo	Earthworm *Perionyx excavatus.*	Inhibition of TCTP protein, suppressed key regenerative proteins, including PCNA (involved in proliferation), Wnt3a (responsible for stem cell activation), and YAP1 (a component of the Hippo signaling pathway). Increased expression of p53 (the cellular stress protein).	Disrupts the regeneration process.	[44]
		Decrease	-	in vitro	Human colorectal carcinoma cell line HCT116 p53^−/−^, human non-small cell lung carcinoma cell line H1299 p53-null.	Inhibition of the p53-MDM2 interaction and reduction in p53 degradation.	Induction of cell cycle arrest and apoptosis.	[59]
Tanshinone IIA (Tan-IIA)	Indirect	Decrease	-	in vitro	Human gastric cancer cells AGS.	Decrease in anti-apoptotic proteins Bcl-xL and Mcl-1 and increase in pro-apoptotic protein Bax, caspase-9, and caspase-3 to induce apoptosis.	Inhibition of the proliferation of AGS cells in a time- and dose-dependent manner.	[1]
Sann-Joong-Kuey-Jian-Tang (SJKJT)	Indirect	Decrease	-	in vitro	Human pancreatic carcinoma cells BxPC-3.	Decreased (Mcl-1, Bcl-2, Bcl-xl, and TCTP levels, but increased the protein expression levels of Bax, caspase-9, TNF-α, caspase-8, and caspase-3.	Induction of apoptosis. Increase in the percentage of cells in the sub-G1 phase and inhibition of proliferation.	[60]
		Decrease	-	in vitro	Human hepatic cellular carcinoma cells Hep-G2.	Time and dose-dependent decrease the protein expression level of Mcl-1, TCTP, but increase the protein expression level of Bcl-xl, Fas, Caspase-8, Caspase-3 and Bax.	Inhibition of proliferation of Hep-G2 cells in a time- and dose-dependent manner.	[61]
PAI-1	-	Decrease	-	in vitro	Human prostate cancer cell line LnCAP.	Downregulation of TCTP and nucleophosmin (B23) protein.	Inhibition of plasmin-driven proteolysis, limiting angiogenesis and metastasis.	[65]

“-” no information.

**Table 4:** The list of Pharmacological agents increasing TCTP levels.

Compound	Effect on TCTP Binding Direct/Indirect	TCTP Level		Level of Evidence	Experimental Model	Potential Molecular Impact of Treatment	Effect of Action	References
		Protein	mRNA					
*** Oxaliplatin or 5-fluorouracil (5-FU)	Indirect	Increase	Decrease	in vitro	Human colon cancer cell line HCT116	Increased TCTP protein levels but decreased TCTP mRNA levels. Increased TCTP protein expression in the presence of 5-FU or oxaliplatin is dependent on mTORC1.	TCTP-siRNA was more sensitive to 5-FU or oxaliplatin. Also, TCTP is indeed able to partially protect HCT116 cells against the cytotoxic effects of 5-FU or oxaliplatin.	[55]
LPS	Indirect	Increase mTCTP and dTCTP	No changes	in vitro	Bone marrow-derived mast cells BMMCs	Increased in mTCTP and dTCTP protein levels, but no change in TCTP transcriptional levels.	Secreted dTCTP increased the expression of IL-8 and pro-inflammatory cytokines in airway epithelial cells.	[67]
M-CSF (Macrophage colony-stimulating factor)	Undetermined	Increase	Increase	in vitro	Mouse peritoneal macrophages	Promotion of synthesis and secretion of TCTP.	Suggested to participate in chronic allergic reactions.	[68]
H_2_O_2_	Indirect	Increase secretion	-	in vitro	Human bronchial epithelial cells HBECs BEAS-2B	Possible triggering of HRF release from bronchial epithelial cells by oxidative stress.	Stimulation of HRF secretion by BEAS-2B cells upon exposure to 50–250 µM H_2_O_2_.	[69]
Dioxin (2,3,7,8-Tetrachlorodibenzo-p-dioxin (TCDD))	Indirect	Increase	Increase	in vitro; in vivo	TT2 embryonic stem (ES) cells; Mouse embryonic fibroblasts (MEFs); Jurkat cell line; BJAB cell line; C57BL/6 mice	Induction of HRF by TCDD mediated through the AhR pathway. Induction of TCTP synthesis and secretion.	Increase in serum histamine levels in mice.	[70]
PMA, forskolin, heavy metals: copper, nickel and cobalt	Undetermined	Increase	Increase	in vitro	Human lung anaplastic carcinoma cell line Calu-6; African green monkey kidney fibroblast-like cell line Cos-7.	Increase in TCTP expression at 24 h in both cell lines, about 2.2–3.2-fold at the mRNA level and 1.6–2.2-fold at the protein level. The highest induction rate: 4.5–5.0-fold at the mRNA level, and 3.5–4.0-fold at the protein level— observed with copper.		[4]
Hypoxia	-	Increase	-	in vitro	Human colon cancer cell line HCT116; LoVo	Hypoxia promotes TCTP expression and secretion through activation of HIF-1α.		[71]

“-” no information; *** The drug is also described in Table 2.

**Table 5:** Summary of Pharmacological agents inhibiting TCTP phosphorylation.

Compound	Effect on TCTP Binding Direct/Indirect	TCTP Level		Level of Evidence	Experimental Model	Potential Molecular Impact of Treatment	Effect of Action	References
		Protein	mRNA					
Onvansertib	Indirect	Decrease pTCTP	-	in vitro	Head and neck squamous cell carcinoma (HNSCC) cell lines: CAL33, CAL27.	Inhibition of Plk1 and reduction phosphorylation of TCTP.	Onvansertib induced mitotic arrest, chromosomal abnormalities, and polyploidy, leading to apoptosis in HNSCC cells.	[72]
		Decrease pTCTP	-	ex vivo; in vitro	18 patients with a confirmed diagnosis of Acute myeloid leukemia (AML); human promyelocytic leukemia cell line HL-60	Decrease in the phosphorylation of the PLK1 substrate TCTP following inhibition of PLK1.	Reduction in pTCTP by plasma samples taken during treatment with Onvansertib, but not by plasma samples in which onvansertib was not detected; pTCTP inhibition was observed using plasma from all dose levels (12–60 mg/m^2^) with greater pTCTP inhibition at higher doses.	[73]

“-” no information.

## Data Availability

The original contributions presented in this study are included in the article. Further inquiries can be directed to the corresponding author.
